# Synergistic antitumor activity of histone deacetylase inhibitors and anti-ErbB3 antibody in NSCLC primary cultures via modulation of ErbB receptors expression

**DOI:** 10.18632/oncotarget.7195

**Published:** 2016-02-04

**Authors:** Chiara Ciardiello, Maria Serena Roca, Alessia Noto, Francesca Bruzzese, Tania Moccia, Carlo Vitagliano, Elena Di Gennaro, Gennaro Ciliberto, Giuseppe Roscilli, Luigi Aurisicchio, Emanuele Marra, Rita Mancini, Alfredo Budillon, Alessandra Leone

**Affiliations:** ^1^ Experimental Pharmacology Unit, Istituto Nazionale Tumori Fondazione G. Pascale - IRCCS, 80131 Naples, Italy; ^2^ Scientific Direction, Istituto Nazionale Tumori Fondazione G. Pascale - IRCCS, 80131 Naples, Italy; ^3^ Takis s.r.l., University, 00161 Rome, Italy; ^4^ Department of Surgery “P.Valdoni” and Department of Clinical and Molecular Medicine, “La Sapienza” University, 00161 Rome, Italy

**Keywords:** NSCLC, HDAC inhibitor, ErbB3, valproic acid, primary tumor cultures

## Abstract

ErbB3, a member of the ErbB family receptors, has a key role in the development and progression of several cancers, including non-small cell lung cancer (NSCLC), and in the establishment of resistance to therapies, leading to the development of anti-ErbB3 therapies.

In this study we demonstrated, in a set of malignant pleural effusion-derived cultures of NSCLC, the synergistic antitumor effect of a histone deacetylase inhibitor (HDACi), such as vorinostat or valproic acid (VPA), in combination with the anti-ErbB3 monoclonal antibody (MoAb) A3. Synergistic interaction was observed in 2D and in 3D cultures conditions, both in fully epithelial cells expressing all ErbB receptors, and in cells that had undergone epithelial to mesenchymal transition and expressed low levels of ErbB3. We provided evidences suggesting that differential modulation of ErbB receptors by vorinostat or VPA, also at low doses corresponding to plasma levels easily reached in treated patients, is responsible for the observed synergism. In details, we showed in epithelial cells that both vorinostat and VPA induced time- and dose-dependent down-regulation of all three ErbB receptors and of downstream signaling. On the contrary, in A3-resistant mesenchymal cells, we observed time- and dose-dependent increase of mRNA and protein levels as well as surface expression of ErbB3, paralleled by down-regulation of EGFR and ErbB2. Our results suggest that the combination of a HDACi plus an anti-ErbB3 MoAb represents a viable strategy that warrants further evaluation for the treatment of NSCLC patients.

## INTRODUCTION

Non-small cell lung cancer (NSCLC) accounts for approximately 85% of all lung cancer cases representing the leading cause of cancer deaths worldwide [1].

Epidermal growth factor receptor (EGFR) tyrosine kinase inhibitors (TKIs) represent the first targeted agents approved for the treatment of NSCLC. However, despite impressive clinical success, particularly in patients harbouring specific somatic activating EGFR gene mutations, almost all of them eventually experience relapse because of acquired drug resistance [2, 3].

In the last years it has been demonstrated that ErbB3, another member of the ErbB family receptors, has a key role in the development and progression of several cancers including NSCLC [4, 5]. In addition, ErbB3 over-expression, which acts as a key node in ligand-induced activation of the ErbB Receptor-PI3K axis, is involved in the mechanism of resistance to EGFR-TKI [6]. Importantly, the EGFR-ErbB3 interdependency has been observed not only in tumor cells harbouring mutationally activated EGFR, but also in tumors with wild-type EGFR, revealing a role for ErbB3 that extends beyond the context of mutationally activated EGFR [6].

Recently, we have generated two ErbB3 monoclonal antibodies (MoAbs), namely A3 and A4, that negatively regulate the ErbB3-mediated signaling pathway, reducing the growth rate of cancer cells from different origins, either *in vitro* [7, 8] or *in vivo* [9]. Specifically, A3, by recognizing the dimerization loop in the second domain in the extracellular region of ErbB3 [10], leads to the internalization and degradation of the receptor and inhibition of its recycling and thus can prevent the ligand-dependent phosphorylation of ErbB3 [9]. Furthermore, we have shown that A3 was able to re-sensitize EGFR-TKI resistant NSCLC cells, leading to inhibition of tumor growth in xenograft models [11].

Histone deacetylase inhibitors (HDACis) represent a class of antitumor agents that, based on the functions of the epigenetic enzymes they regulate, are able to affect multiple genes and pathways and to synergize with diverse anticancer conventional and targeted drugs [12–19]. In squamous cell carcinoma of head and neck (SCCHN) cells, we have recently demonstrated that the clinically approved HDACi vorinostat enhanced the antitumoral effects of the EGFR-TKI gefitinib, by a mechanism depending on the ErbB3 status and on the tumor cell phenotype (epithelial vs. mesenchymal). In details, we showed that vorinostat downregulated the expression of all ErbB receptors in epithelial cells, while in cells which had undergone epithelial to mesenchymal transition (EMT) reverted the mesenchymal phenotype by inducing both E-cadherin and ErbB3 and downregulating vimentin as well as EGFR and ErbB2 [16]. Recently, we have also shown that vorinostat is able to increase the therapeutic efficacy of EGFR-TKIs gefitinib or erlotinib in a panel of NSCLC cell lines by altering redox homeostasis [20].

Valproic acid (VPA) is a generic low-cost anticonvulsant and mood stabilizer, that has been used for over 40 years, that demonstrates HDAC inhibitory activity and anticancer properties [21, 22].

In this study, we investigate the effects of a novel combinatorial strategy based on the use of a HDACi, such as vorinostat or VPA, plus the anti-ErbB3 MoAb, A3, in a set of primary tumor cultures from malignant pleural effusions (MPEs) of NSCLC patients. We demonstrated antitumor synergistic effect of the combination in both 2D and 3D conditions. We also provided evidences that the mechanism underlying the synergistic interaction between the two classes of agents is related to the downregulation of EGFR and ErBB2 and to the differential modulation of ErbB3 based on tumor cell phenotype, epithelial vs mesenchymal, induced by the HDACis.

## RESULTS

### The antiproliferative effect of vorinostat and VPA on primary NSCLC cultures is independent from the basal expression of ErbB receptors and EMT markers as well as from the tumor genetic background

**Figure 1:**
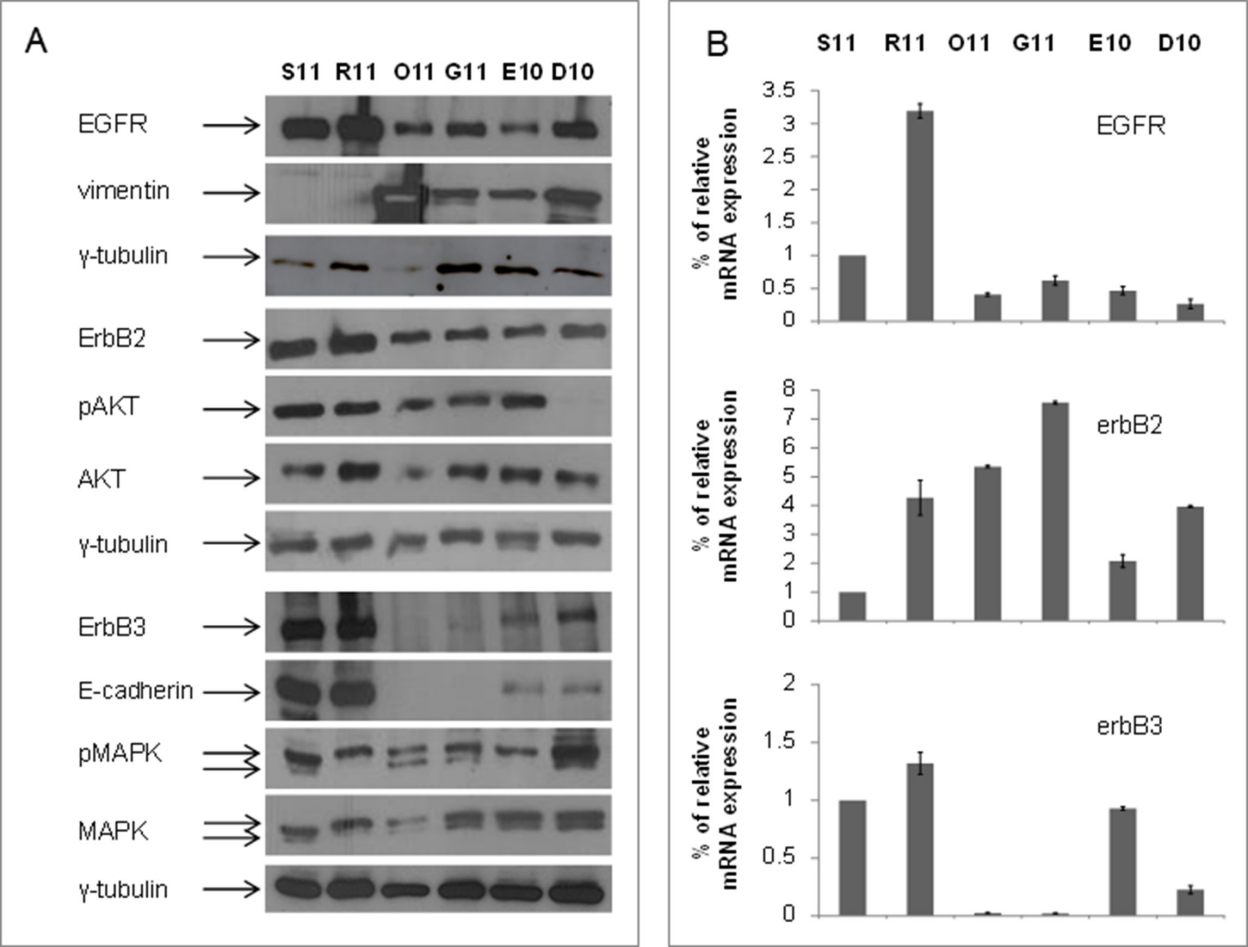
Basal levels of ErbB3 protein and mRNA correlated with the epithelial marker E-cadherin in a set of malignant pleural effusions (MPE) cells from NSCLC patients (**A**) Protein lysates from S11, R11, O11, G11, E10 and D10 cells at basal levels, were resolved by SDS-PAGE and immunoblotted with anti-EGFR, ErbB2, ErbB3, E-cadherin, vimentin, pAKT/AKT and pMAPK/MAPK antibodies. γ-tubulin was used as protein loading control. (**B**) ErbBs mRNA levels, derived from MPE cells, were analyzed by qRT-PCR, after 24 h from seeding and β-actin was used as housekeeping control gene.

First of all, we evaluated the basal levels of ErbB receptors and downstream pathways as well as of the markers involved in EMT, in a panel of primary tumor cultures derived from MPEs of patients affected by NSCLC [23]. We identified ErbB3 as a clear epithelial marker, since cells expressing high levels of ErbB3 protein (S11 and R11) also showed high expression of conventional epithelial marker E-cadherin and no expression of the mesenchymal marker vimentin (Figure 1A). Conversely, cells with absence or faint expression of ErbB3 (O11 and G11), similarly to the non-tumorigenic human fibroblast cell line BJhTERT, demonstrated high levels of vimentin but did not express E-cadherin (Figure 1A and Supplementary Figure 1A). E10 and D10 cells showed an intermediate phenotype expressing both vimentin and E-cadherin and low levels of ErbB3. In all cell lines studied, ErbB3 protein levels correlated also with mRNA expression (Figure 1B). Stimulation of cells by heregulin, an ErbB3-ligand, induced an increase of ErbB3 activity only in S11 cells, but not in O11 cells, confirming the absence of a functional ErbB3 receptor on this latter cell line (Supplementary Figure 1B). Notably, all primary cultures expressed different basal levels of EGFR and ErbB2 as well as of downstream effectors, such as pAKT/AKT or pMAPK/MAPK (Figure 1A).

The genetic background of the six cell cultures varies and, at least in part, recapitulates the heterogeneity of NSCLC. The epithelial R11 and S11 cells have the EGFR exon 19 activating deletion, the latter cell line expressing also the T790M mutation while the mesenchymal G11 and O11 cells and the intermediate E10 and D10 cells are EGFR-wt. Moreover, E10, O11 and D10 cell lines show a K-Ras mutation (Table 1).

**Table 1:** Malignant pleural effusion (MPE) cell lines genetic backgrounds and antiproliferative effects of vorinostat and valproic acid (VPA)

Cell Lines	EGFR status^a^	K-Ras status	Additional features	Vorinostat IC_50_ (μM) 72 h (± S.D)	VPA IC_50_ (mM) 72 h (± S.D)
**S11**	Ex19 del15bp T790M	wt	p53-mut (S241F)	1.28 ± 0.4	3.4 ± 0.1
**R11**	Ex19 del15bp	wt	-	0.93 ± 0.3	1.93 ± 0.4
**G11**	wt	wt	-	5.15 ± 1.3	9.3 ± 1.2
**O11**	wt	p.Q61H	PI3K-mut STK11-mut (Q37*)	1 ± 0.3	1.96 ± 0.8
**D10**	wt	p.G12V	-	7 ± 1.7	12.73 ± 1.5
**E10**	wt	p.Q61H	MET-T1010I SNP	4.87 ± 2.3	6.9 ± 1.2

a For all cell lines information regarding mutational status were previously reported [11].

We next investigated the antiproliferative effects exerted by vorinostat and VPA on all cell lines and demonstrated that R11 and O11 cells are the most sensitive to both agents, S11 are more sensitive to vorinostat than to VPA, while G11, D10 and E10 cells are resistant to both HDACis (Table 1).

### Vorinostat and VPA modulate ErbB receptors expression in primary NSCLC cultures

**Figure 2:**
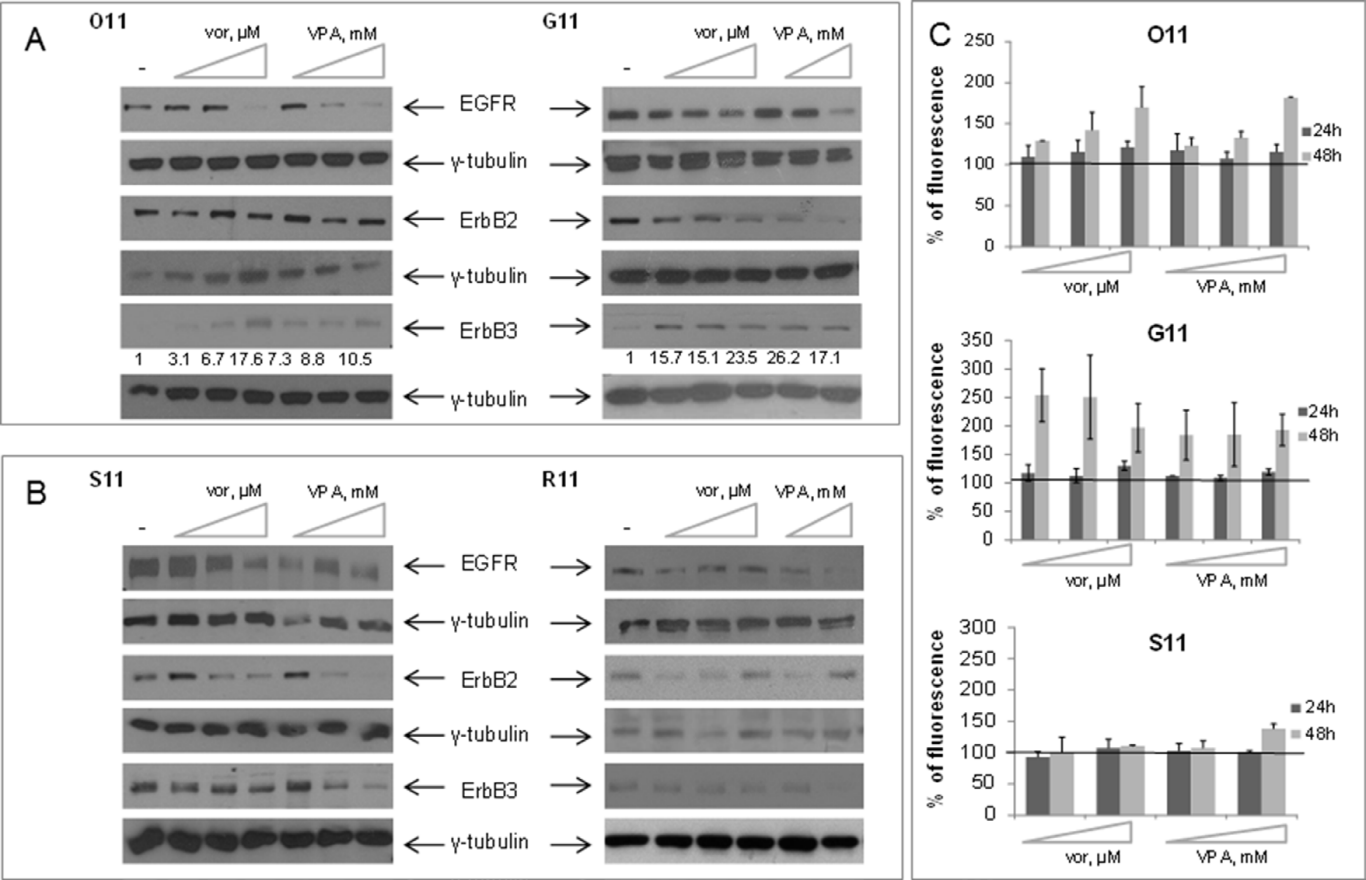
Vorinostat and VPA modulated the expression of ErbB tyrosine Kinase receptors in MPE cell lines, based on cell phenotype Western blot analysis of ErbB receptors was performed on cell lysates obtained from O11 and G11 mesenchymal cells (**A**) and from S11 and R11 epithelial cells (**B**), untreated or treated with increasing doses of either vorinostat or VPA. Cell lysates were collected at 24 h (O11 and S11) or 48 h (G11 and R11) after HDACis treatment. γ-tubulin was used as protein loading control. Treatment doses as follows: O11 cells, vorinostat 0.15–0.3–0.85 μM and VPA 0.55–1–2.8 mM; S11 cells, vorinostat 0.2–1.5–3.5 μM and VPA 1–4–8 mM; G11 cells, vorinostat 1.5–2.3–4.7 μM and VPA 1.5–4.6 mM; R11 cells, vorinostat 0.13–0.4–0.9 μM and VPA 0.2–0.5 mM. (**C**) Approximately 1 × 10^5^ cells/well were cultured in 6-well plates and incubated with increasing doses of either vorinostat or VPA, for 24 h and 48 h. ErbB3 surface expression, assessed by indirect immunofluorescence, was expressed as% of control. Treatment doses as follows: O11 cells, vorinostat 0.15–0.3–0.85 μM and VPA 0.55–1–2.8 mM; G11 cells, vorinostat 1–4–16 μM and VPA 2–8–16 mM; S11 cells, vorinostat 1.5–3.5 μM and VPA 4-8 mM.

Based on our previous results in SCCHN cells [16], we next investigated the effects of both vorinostat and VPA on ErbBs expression in the two mesenchymal O11 and G11 cells and in the two fully epithelial S11 and R11 cells. As shown in Figure 2, in the two mesenchymal cells, expressing basal undetectable levels of ErbB3, we demonstrated a dose-dependent increase of ErbB3 and a downregulation of both EGFR and ErbB2, induced by both vorinostat and VPA (Figure 2A). Conversely, in the two epithelial S11 and R11 cells, expressing high basal levels of EGFR, ErbB2, and ErbB3, the treatment with either vorinostat or VPA clearly reduced the protein expression of all three ErbB receptors (Figure 2B). Notably, in O11 and G11 cells we also showed that the increase of ErbB3 protein levels correlated with increased cell surface expression within 24 h of treatment with a pick after 48 h. In S11 cells ErbB3 surface expression was apparently unchanged up to 48 h (Figure 2C).

**Figure 3:**
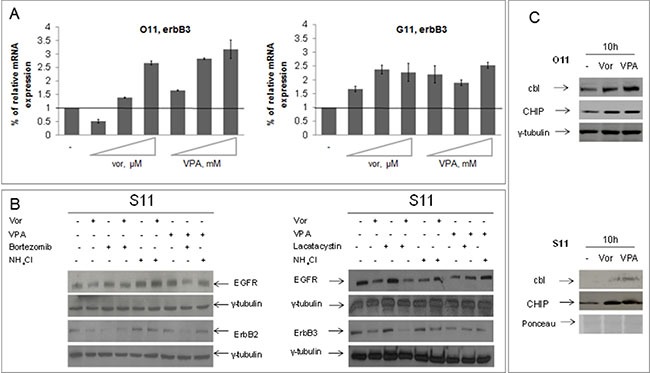
Vorinostat and VPA regulated at different levels ErbB receptors expression in mesenchymal and epithelial primary cultures (**A**) O11 and G11 mesenchymal cells were untreated or treated with increasing doses of vorinostat or VPA for 24 h and erbB3 mRNA levels were assessed by qRT-PCR, as described in Materials and Methods Section. β-actin mRNA levels were used as housekeeping control gene. Treatment doses as follows: on O11 cells, vorinostat 0, 15–0, 3–0, 85 μM and VPA 0, 55–1–2, 8 mM; on G11 cells, vorinostat 1, 5–2, 3–4, 7 μM and VPA 2, 7–4, 6–8, 95 mM. (**B**) S11 epithelial cells were treated with HDACis (vorinostat 1 μM and VPA 0.5 mM) and/or lactacystin 5 mM or bortezomib 10 nM or NH_4_Cl 10 mM at 24 h. Cell extracts were resolved by SDS–PAGE and immunoblotted with anti-ErbBs. γ-tubulin immunoblotting indicated the equal loading of samples in each lane. (**C**) Protein lysates from O11 (upper panel) and S11 (bottom panel) cells, collected after 10 h of both HDACis treatment, were resolved by SDS-PAGE and immunoblotted with anti-cbl and anti-CHIP antibodies. γ-tubulin or ponceau staining were used as protein loading control.

We also showed that the HDACi-induced ErbB3 expression in O11 and G11 cells is dependent on the upregulation of erbB3 mRNA levels (Figure 3A), while the downregulation of EGFR and ErbB2 in these two cell lines, and of all ErbB receptors in epithelial R11 and S11 cells, is related to protein degradation (Figure 3B). Indeed, in epithelial S11 cells, we evaluated the impact of either proteasome inhibitors (lactacystin or bortezomib) or lysosome neutralizing agent ammonium chloride (NH_4_Cl) on vorinostat or VPA-dependent ErbB receptors degradation. We demonstrated that the downregulation of all ErbB receptors, induced by either one of the two HDACis, was completely reverted by concomitant treatment with NH_4_Cl, and, partially by bortezomib (Figure 3B-left panel) or lactacystin treatment (Figure 3B-right panel). Similarly, HDACi-induced EGFR downregulation in O11 cells appeared related with both proteasomal and lysosomal degradation (Supplementary Figure 2).

In order to confirm that EGFR and ErbB2 downregulation, observed in all cell lines upon HDACis treatment, was due to protein degradation, and to further explore the mechanism of this effect, we evaluated the expression level of cbl, an E3-ubiquitine ligase which mediates the degradation of EGFR, and of CHIP, a chaperone-binding ubiquitin ligase (CHIP) that shorten the half-life of ErbB2. We demonstrated increased expression of both ubiquitin ligases after 10 h of treatment with either vorinostat or VPA in both O11 and S11 cells (Figure 3C).

**Figure 4:**
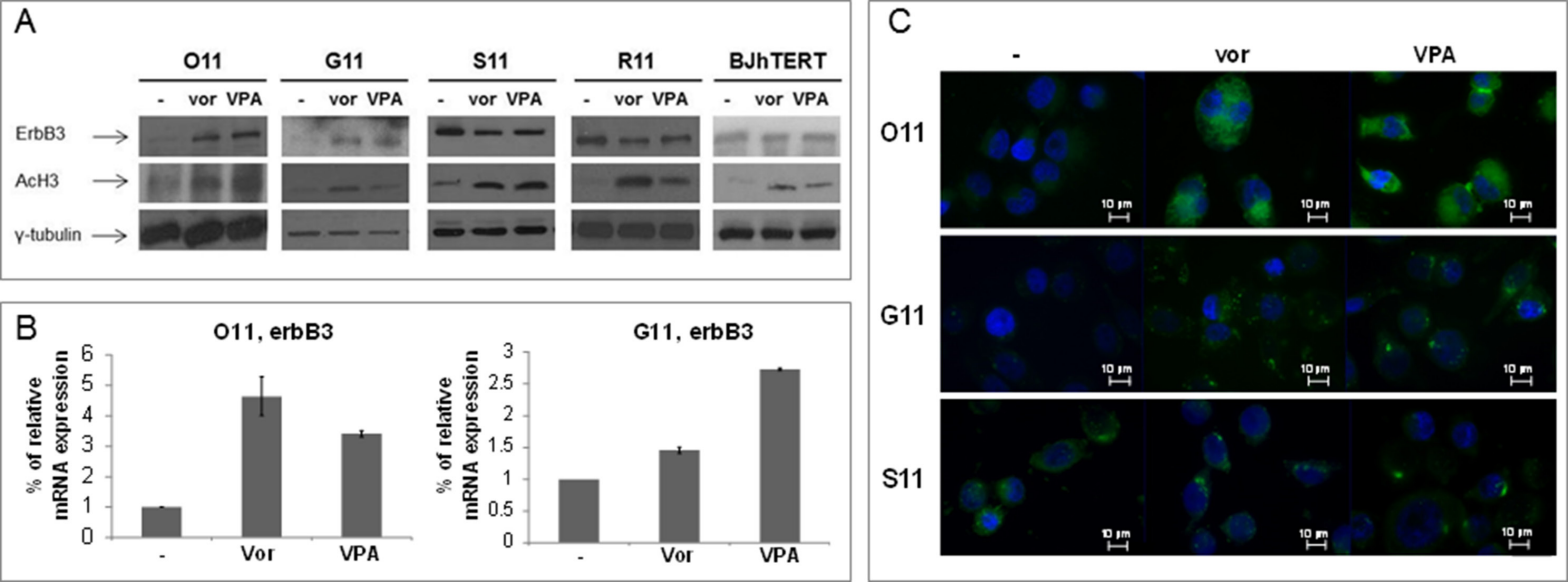
Vorinostat and VPA modulated ErbB3 expression at low doses, corresponding to plasma levels easily reached in patients MPE cells and normal fibroblast (BJhTERT) cell line were treated with vorinostat and VPA at 1 μM and 0.5 mM, respectively. (**A**) Equal amount of proteins, collected at indicated times (24 h for O11 and BJhTERT; 48 h for G11 and R11; 72 h for S11) was resolved on SDS-PAGE and immunoblotted with anti-ErbB3 antibody. γ-tubulin immunoblotting ensured the equal loading of samples in each lane, while acetylation of histone H3 confirmed the activity of HDACis. (**B**) ErbB3 mRNA levels were analyzed by qRT-PCR at 8 h treatment. β-actin was used as housekeeping control gene. (**C**) ErbB3 protein expression was shown by fluorescent microscopy at 48 h in G11 cells and at 24 h in both O11 and S11 cells.

We also confirmed in O11 and G11 a strong upregulation of ErbB3 protein and mRNA levels, evaluated by western blotting, qRT-PCR and immunofluorescence (Figure 4A–4C), upon treatment with low doses of vorinostat or VPA (vorinostat: 1 μM and VPA: 0.5 mM), easily reached in the plasma of treated patients [24, 25]. In S11 and R11 cells, we confirmed at low doses of both HDACis the downregulation of ErbB3, evaluated by western blotting (Figure 4A) and immunofluorescence (Figure 4C). Notably, in the non-tumorigenic human fibroblast BJhTERT cells, although increased histone-H3 acetylation was evident after 24 h, we did not observe any change of ErbB3 expression (Figure 4A).

All together these data demonstrated that clinically reached low doses of either vorinostat or VPA, depending on cell phenotype and/or basal expression, differentially modulated the expression of ErbB3 in NSCLC primary cultures but not in normal non-tumorigenic cells.

### Synergistic antitumor effect of vorinostat or VPA in combination with the anti-ErbB3 MoAb A3 in 2D and 3D primary NSCLC cultures

**Figure 5:**
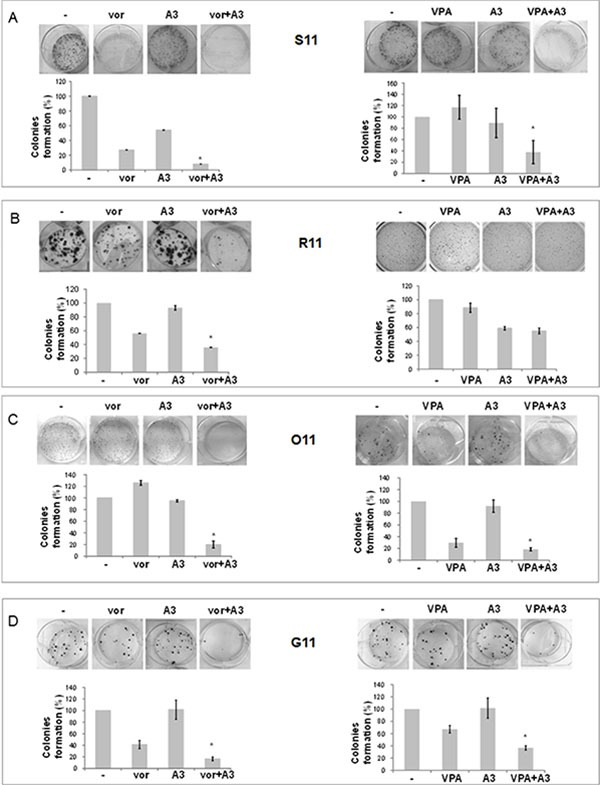
Simultaneous treatment with HDACi/moAb A3 induced a synergistic inhibition of MPE cell survival, independently of cells phenotype (**A**–**D**) Clonogenic assays were performed on S11 (A), R11 (B), O11 (C) and G11 (D) cells, plated at 300 cells/well in 6 well/plates treated and untreated with different concentrations of A3 and vorinostat or VPA. After 10 (O11), 14 (S11 and G11) and 13 (R11) days, colonies number was visualized and counted by SRB or crystal violet colorimetric assays. Treatment doses as follows: S11 cells, vorinostat 1 μM, VPA 0.5 mM, and A3 1 μg/ml; R11 cells, vorinostat 0.75 μM, VPA 0.5 mM and A3 1 μg/ml; O11 cells, vorinostat: 0.25 μM, VPA 0.8 mM and A3 1 μg/ml; G11 cells, vorinostat 1 μM, VPA 0.5 mM and A3 1 μg/ml. (A–D) Colonies formation was expressed as% of control. Statistical analysis in MPE cells for combination vs single agent treatment is reported (*p < 0.05 combination vs single agent).

On the bases of HDACi-mediated ErbB3 modulation, we next investigated the antitumor capability of low doses of vorinostat or VPA in combination with the novel anti-ErbB3 MoAb, A3 [9], in the ErbB3 expressing S11 and R11 cells and in the non-ErbB3 expressing O11 and G11 cells. Specifically, we performed clonogenic assays showing a strong synergistic reduction in colonies formation in the combination setting with both HDACis compared to single agent treatments or control untreated cells, and in all cell lines (Figure 5A–5D). Similar effects were obtained by a soft-agar assay (Supplementary Figure 3) and with sequential treatment of HDACi followed by A3 antibody (Supplementary Figure 4). Notably, the antiproliferative effect of A3 by itself appeared to be evident only in the epithelial S11 and R11 cells compared to mesenchymal O11 and G11, accordingly with previous data [11] and basal level of ErbB3.

Next, because 3D cultures better recapitulate tumor growth complexity compared to 2D monolayers conditions, we tested HDACi/A3 combination also on 3D tumor spheroid derived from our primary NSCLC cultures.

**Figure 6:**
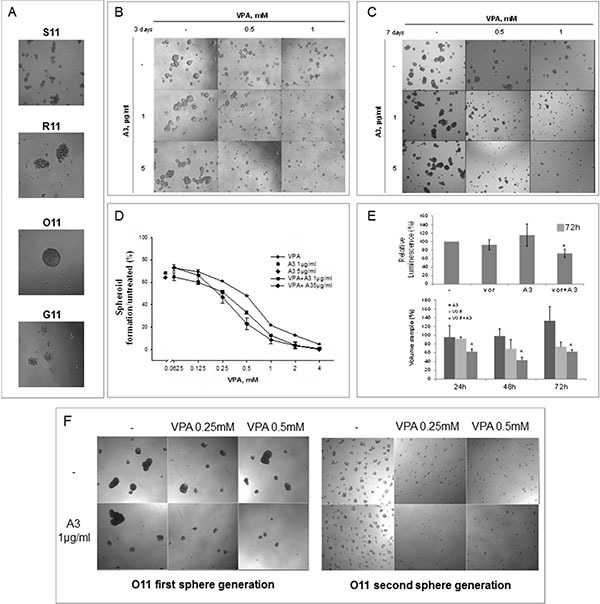
Simultaneous HDACi/A3 treatment inhibited synergistically O11 spheroids formation and propagation (**A**) 40000 cells/ml, either epithelial or mesenchymal, were plated in sphere medium and imaged after 3 days by contrast microscopy (10X). (**B**–**F**) Sphere forming assay was performed on O11 cells, untreated or treated with increasing doses of VPA and/or A3 at 1 μg/ml or 5 μg/ml. At indicated doses spheroids images were taken after 3 days (B) and 7 days (C) treatment by contrast microscopy (10X). (D) The spheroids were counted and spheroids cell formation was expressed as% of control, after 3 days of treatment. (E) In 3D cell viability assay O11 spheroids were treated with A3 5 μg/ml and/or vorinostat 1 μM for 72 h. Spheroids viability was assessed by luminescence assay (upper panel) or by volume reduction measurements (bottom panel) as described in Material and Method section. Values were expressed as% of control. (F) First-generation of sphere forming assay was performed on O11 cells, untreated or treated with increasing doses of VPA and/or A3 at 1 μg/ml and images were taken after 3 days (F-left panel). O11 spheroids, survived to the first-generation treatment, were disaggregated and plated again to form second-generation spheroids without any additional treatment. Second-generation spheroids were imaged after 3 additional days by contrast microscopy (F-right panel).

First of all, we showed that O11 mesenchymal cells were able to form big spheroids already at 3 days, compared to other cell cultures (Figure 6A). Thus, we considered them as the best model to study the impact of our therapeutic combination on tumor spheroids. Consistently with our 2D clonogenic assay, we demonstrated that the VPA/A3 combination, compared to single agent treatments, strongly affected tumor cell growth by inhibiting first-generation spheroids formation (Figure 6B–6D), viability (Figure 6E-upper panel) and volume (Figure 6E-bottom panel). In details, a spheroids number reduction was observed at 3 days (Figure 6B and 6D) and up to 7 days (Figure 6C) in cells treated only once with each drug, suggesting that a single-time combination treatment is enough to inhibit spheroids formation.

Finally, an assay on second-generation spheroids allowed us to explore the effect of the combination on putative tumor-initiating cells (Figure 6F). Concordantly to previous results, a strong reduction on spheroids formation was observed again on first-generation spheroids after 3 days of treatment by VPA/A3 combination (Figure 6F-left panel). Thereafter, when O11 survived spheroids, considered enriched in putative initiating tumor-stem cells, were disaggregated and plated again to form second-generation spheroids without any additional treatment, we confirmed the synergistic reduction of spheroid formation (Figure 6F-right panel), suggesting that a single-time treatment is able to affect both mature and putative tumor initiating cells.

All together these data suggested that the modulation of ErbB receptors and particularly of ErbB3 by a HDACi, such as vorinostat or VPA, lead to a clear synergistic antitumor effect in combination with an anti-ErbB3 monoclonal antibody such as A3. Moreover this new therapeutic strategy is able to have a strong impact on putative tumor initiating cells that usually are not responsive to standard treatment.

### Vorinostat or VPA in combination with the ErbB3 MoAb A3 downregulates ErbB3 downstream AKT and MAPK signaling and induces DNA-damage and apoptosis

**Figure 7:**
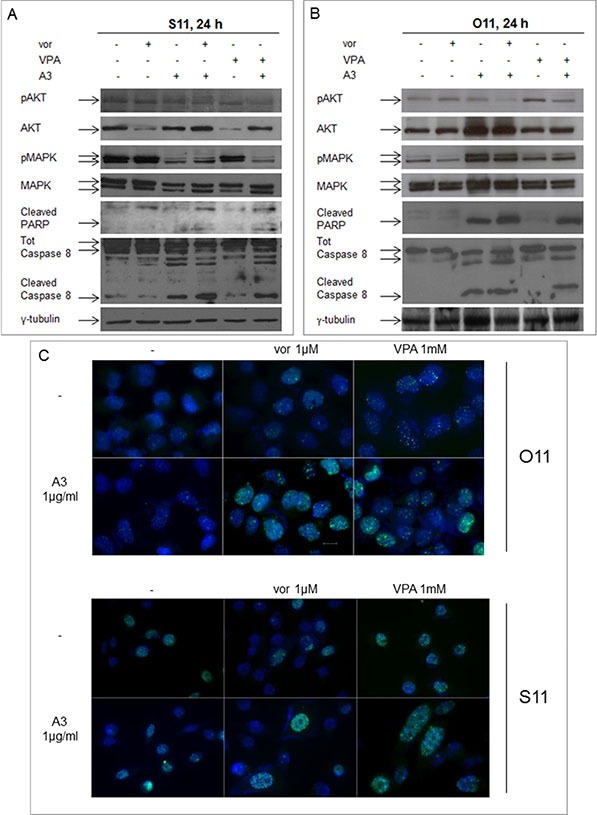
Synergistic modulation of mitogenic signaling as well as of DNA-damage and apoptosis by HDACi and A3 combination treatment in MPE cells Protein lysates from S11 (**A**) and O11 (**B**) cells, untreated or treated for 24 h with vorinostat 1 μM or VPA 0.5 mM and/or A3 25 μg/ml, were immunoblotted with pAKT/AKT, pMAPK/MAPK cleaved PARP and cleaved caspase 8 antibodies. γ-tubulin protein expression was used as protein loading control. (**C**) Cells treated with or without HDACi and/or A3 at the indicated concentration, were collected 24 h after drugs treatment. Then, cells were stained for γH2AX (green) and DAPI for nuclei (blue) and observed by microscope, as indicated in Materials and Methods section.

To investigate at the molecular level the HDACis/A3 synergistic interaction, we evaluated ErbB3-downstream AKT and MAPK signaling in S11 (Figure 7A) and O11 cells (Figure 7B). We observed in both cell lines a complete deactivation or strong reduction of AKT activity in the combination setting, with either vorinostat or VPA, compared to single agent treatments or control untreated cells. Conversely, a clear downregulation of MAPK activity in the combination setting was observed in S11 but not in O11 cells, probably due to K-Ras mutation in the latter cell line (Table 1).

Finally, in both cell lines, HDACi/A3 combination also induced a pro-apoptotic effect as shown by poly-(ADP-ribose)-polymerase (PARP) and caspase 8 cleavage (Figure 7A and 7B) as well as DNA-damage effect, highlighted by the increase in nuclear γH2AX foci formation, a marker of double strand breaks (Figure 7C).

All together these data confirmed the synergistic interaction of the HDACi/A3 combination.

## DISCUSSION

In the present study we demonstrated for the first time the synergistic antitumor effect between a HDACi, such as vorinostat or VPA, and the anti-ErbB3 MoAb A3, in a set of NSCLC primary tumor cultures. The synergistic antitumor effect was demonstrated in cells expressing different mutational status of EGFR or K-Ras, different basal levels of ErbB receptors as well as either epithelial or mesenchymal phenotype. We showed that both vorinostat and VPA were able to downregulate EGFR and ErbB2 expression in all our primary NSCLC cultures, as previously reported by us and others in different tumor cell models, including NSCLC cell lines [16, 20, 26–29]. We also showed that ErbB3, which appears to be a clear epithelial marker paralleling E-cadherin expression, was downregulated in fully epithelial primary NSCLC cultures. Conversely, in mesenchymal vimentin-expressing NSCLC cultures, both vorinostat and VPA induced ErbB3 expression and enhanced ErbB3 localization to the plasma membrane. This latter event is reported as associated to enhanced ErbB3 biological activity [30, 31]. Notably, these effects were confined to cancer cells and were observed also at low doses of either vorinostat or VPA, corresponding to plasma levels of each drug easily reached in treated patients. Downregulation of EGFR, ErbB2 and ErbB3, induced by vorinostat and VPA, was due to protein degradation, either by the proteasome or the lysosomes, confirming our previous observations [16]. In agreement to our data, previous studies have demonstrated that ErbB3 could be degraded not only by proteasome ubiquitination, but also by internalization of ErbB3 into the lysosome, leading to the inhibition of cell surface recycling [32–35]. Although either downregulation of EGFR and ErbB2 or increased ErbB3 expression upon treatment with HDACi have been reported before in tumor cells and particularly in NSCLC cell lines [16, 29, 36–40], to our knowledge this is the first study reporting these effects in primary cultures.

Moreover, the HDACi-mediated early upregulation of the ubiquitine-ligases CHIP and cbl are consistent with their involvement in ErbB2 and EGFR downregulation, respectively. Interestingly, loss of cbl expression has been demonstrated in lung cancer patients compared with their adjacent normal epithelium [41]. In addition, although a recent study also showed that the upregulation of cbl, induced by HDACi, contributed to growth inhibition and apoptosis as well as EGFR down-regulation in NSCLC cells [41], to our knowledge our study is the first to demonstrate CHIP modulation by a HDACi.

However, the crucial finding of this study was the demonstration of the efficacy of a novel treatment combination strategy in NSCLC models, based on the use of a HDACi, plus an anti-ErbB3 MoAb such as A3, previously generated by our group [9, 23]. Indeed, simultaneous exposure to the clinically approved vorinostat or the safe and well known anti-epileptic VPA with A3 showed strong antiproliferative and pro-apoptotic effect as well as DNA-damage.

Interestingly, we have recently demonstrated that vorinostat can improve the therapeutic efficacy of EGFR-TKIs in NSCLC models, including O11 cells, by a mechanism related to increased oxidative stress and a consequent DNA-damage [20]. Although in the present study we have not explored oxidative stress pathway, we can hypothesize that the concomitant treatment with a HDACi and an anti-ErbB3 MoAb induced DNA-damage by altering oxidative homeostasis and/or by inhibiting survival/DNA-repair pathways regulated by ErbB receptors. In this regard, it has been recently shown that ErbB3 may play an important role in response to radiation therapy and blocking its activity in combination with radiation increased DNA-damage being of therapeutic benefit in human tumors [42].

Notably, the synergistic antitumor effect was also observed on 3D tumor spheroid cultures, where a reduction in cell proliferation was also evident up to 7 days culture and after a single treatment with the HDACi/A3 combination, suggesting that a single priming is sufficient to induce some alterations that can impact on spheroids formation. Furthermore, similar results were obtained even on untreated second-generation spheroids derived from survived treated first-generation, highlighting the capability of the combination to affect also the putative initiating tumor-stem cells population. To date, although it has been demonstrated a role for epigenetic modifications in the reprogramming of cancer stem cells, it is still unknown how HDACis are able to influence initiating tumor-stem cells fate [43, 44]. Because we have previously described that the MPE-derived cultures became more undifferentiated when shifted from adherent cells to spheroids [23, 45], the epigenetic reprogramming exerted by HDACis on spheroids themselves could result in a crucial mechanism to limit their aggressiveness.

**Figure 8:**
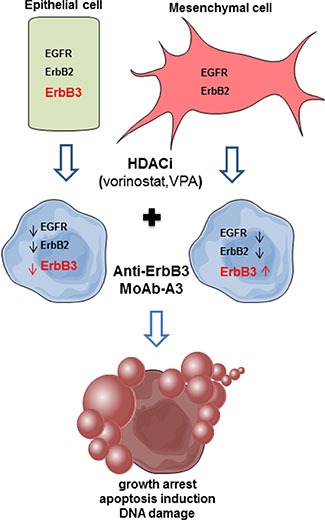
Proposed model of combination treatment mechanism of action In epithelial cells, expressing all ErbB receptors, we propose that the synergistic antiproliferative effect between a HDACi and A3 is due to the ability of the HDACi to reduce ErbB3, the target of the A3 MoAb itself, together with both EGFR and ErbB2, crucial partners for ErbB3. In the A3-resistant mesenchymal cells, the HDACi, by inducing ErbB3, lead them to become dependent to ErbB3 signaling and therefore susceptible to A3 MoAb. Moreover, the concomitant reduction of EGFR and ErbB2 expression by the HDACi, and the ErbB3-blocking, by A3, trigger the observed antiproliferative effect.

The mechanism of the observed synergism between HDACi and anti-ErbB3 MoAb appears to be multifactorial (Figure 8). We can speculate that, the HDACi-induced expression of ErbB3 in mesenchymal cells, that do not express the receptor and are indeed resistant to the anti-ErbB3 MoAb A3 [11], could lead them to become dependent to the signaling pathway mediated by ErbB3 receptor and, therefore, susceptible to the action of the anti-ErbB3 monoclonal antibodies. In other words, by treating the cells with HDACi we induced a “target prioritization” [46], increasing the sensitivity to ErbB3-inhibitor and avoiding the need of a wide inhibition of multiple survival signals, normally needed to control solid tumors. Indeed, induction of ErbB3, which is mainly involved in the activation of intracellular pro-survival PI3K/Akt/mTOR axis, can lead to couple, as previously reported, EGFR to the PI3K/AKT avoiding other escaping pathways [46]. This observation was reinforced by data demonstrating a strong downregulation of AKT activity, the prominent ErbB3 downstream-target, by the combination in O11 cells, while MAPK-signaling was weakly affected. Concomitantly, the further inhibition of EGFR and ErbB2 leads to an absence of ErbB3 partners and thus to a reduction of tumorigenic signaling.

Conversely, in epithelial cells, where ErbB3 is expressed and could be a driver-factor, the synergistic antiproliferative effect between HDACi and A3 could be due to the capability of the HDACis to synergistically reduce ErbB3, the target of the monoclonal antibody itself, paralleled by the reduction of both EGFR and ErbB2, the crucial partners of the kinase-dead ErbB3 receptor. Indeed, in these cells we observed a strong reduction of both AKT and MAPK signaling. Similarly, it has been demonstrated that the down-regulation of ErbB3 expression, and of its downstream pathways, by the HDACi SNDX-275, is a crucial mechanism to overcome the resistance to the anti-ErbB2 antibody trastuzumab in an ErbB2-overexpressing breast cancer model [47, 48].

Since ErbB3 is a kinase-dead receptor, the only strategy to inhibit its activity is based on monoclonal antibodies, currently under preclinical and clinical evaluations [30, 49, 50]. Unfortunately, anti-ErbB3 antibodies may not result to be highly efficacious as single agents, because of tumor cells ability to escape from ErbB3 inhibition by activation of EGFR and/or ErbB2 [30]. Indeed, we have previously suggested that anti-ErbB3 antibodies could be effective as single agent only in a subset of NSCLC xenografts expressing surface levels of the receptor above a certain threshold, that we showed to correspond to increased AKT pathway activation levels [11]. We have also demonstrated that combination therapy with anti-ErbB3 monoclonal antibodies and EGFR-TKIs potently inhibits NSCLC cells [11]. Similarly, different bispecific antibodies targeting EGFR/ErbB3 or ErbB2/ErbB3 have been developed with the aim to obtain stronger antitumor effects [30, 51–53]. In the present study, we suggested an additional approach, based on the ability of the HDACis vorinostat and VPA to enhance the A3 anti-tumor activity, and provided evidences, for the first time, that the HDACi/A3 combination represents a viable strategy that warrants clinical investigation for the treatment of NSCLC patients, independently of EGFR status or other molecular features.

## MATERIALS AND METHODS

### Materials

Vorinostat and valproic acid (VPA) were provided by Merck & Co., Inc. (Rahway, NJ, USA) and by Enzo Life Sciences (Florence, Italy), respectively. A3 monoclonal antibody was generated as previously described [9]. Stock solutions of vorinostat were prepared in DMSO, while VPA and A3 were prepared in sterile water. Aqueous ammonium chloride (NH_4_Cl) and Lactacystin were obtained from J.T. Baker (Deventer, Holland) and from Calbiochem (Milan, Italy), respectively. Bortezomib and recombinant Human HRG were acquired from R & D System Company (Minneapolis, USA).

Primary antibodies were purchased as follows: EGFR, ErbB2, pErbB3, pAKT, AKT, pMAPK, MAPK, cbl, CHIP, acetyl-H3 and cleaved caspase 8 antibodies from Cell Signaling Technology, Inc. (Boston, MA, USA), ErbB3 and γ-tubulin antibodies from Santa Cruz Biotechnology, Inc. (San Jose, CA, USA), vimentin from DakoCytomation (Glostrup, Denmark), E-cadherin antibody from Abcam (Cambridge, England), cleaved PARP [Poly(ADP-ribose) Polimerase] from BD Pharmingen (Becton Dickinson, San Josè, CA, USA), and γH2AX from Millipore (Billerica, MA, USA).

Secondary antibodies anti-rabbit, anti-goat and anti-mouse were purchased from Abcam (Cambridge, England), anti-rabbit-FITC antibody from Santa Cruz Biotechnology Inc. (San Jose, CA, USA), while anti-rabbit Alexa Fluor 488 was obtained from Life Technologies (Gaitherburg, MD, USA). Enhanced chemiluminescence (ECL) immunodetection reagents were from GE Healthcare (Milan, Italy).

All media, serum, antibiotics and glutamine were from Cambrex Bio Science (Verviers, Belgium). Fetal bovine serum (FBS) was purchased from Gibco (Gaitherburg, MD, USA).

### Cell viability assay

Primary tumor cell cultures derived from a malignant pleural effusion of lung adenocarcinoma patients were previoulsy described [23]. Normal fibroblasts BJhTERT were purchased from American Type Culture Collection (Rockville, MD, USA). Cell lines were grown in the appropriate medium (RPMI for all primary cell lines and DMEM for BJhTERT) supplemented with 10% FBS, penicillin (50 units/ml), streptomycin (500 μg/ml) and 2 mM glutamine, in a humidified atmosphere of 95% air and 5% CO2 at 37°C.

Cell proliferation was assessed in 96-well plates and measured, after cells were treated with dose-escalation of vorinostat or VPA for 72 h, by a spectrophotometric dye SRB-based incorporation assay, as described previously [54].

### Western blotting

Cells were grown and treated with drugs at the indicated concentrations. For HRG-stimulation experiment, cells, seeded in 100 mm dishes, were starved O.N. and the day after treated with drugs as indicated in the figure. At indicated time, cells were collected and equal amounts of protein were resolved and immunoblotted with specific antibody as described previously [18]. Densitometric analysis was performed by ImageJ software (National Institute of Health, USA).

### Real-time PCR

Total RNA was isolated from cells, using Trizol^®^ total RNA isolation reagent (Gibco, Gaitherburg, MD, USA), according to the manufacture's recommendations. cDNA for qRT-PCR analyses was synthesized with the QuantiTect Reverse Transcription Kit (Qiagen, Valencia, CA, USA). For erbB receptors qRT-PCR, cDNA was analyzed using the appropriate Taqman probes by an 7900 HT Sequence Detection System (Applied Biosystems, Foster City, CA, USA). Gene expression modulation was meseaured by the −2^ΔΔCT^ method [55] and normalized to β-actin levels as endogenous control.

### Indirect immunofluorescence assay

#### Immunofluorescence by flow cytometer

Cells, plated in 6 wells plate at 150000 cell/well, were treated, for 24 h or 48 h, with vorinostat and VPA at increasing doses as indicated in figure legend, and then incubated with primary anti-ErbB3 antibody for 30 min at 37°C. After three washes with PBS, cells were incubated with anti-rabbit FITC in the same conditions and evaluated by Becton Dickinson FACSCalibur flow cytometer. Data were analyzed using the Becton Dickinson CellQuest Pro software package.

#### Immunofluorescence by microscopy

Cells, plated on slides in 12 wells plate at 30000 cell/well, were treated with drugs as indicated in figure legends. Then cells were fixed in 4% paraformaldehyde (20 min at RT), blocked by 0.2% PBS/BSA solution (5 min at RT) and incubated with primary anti-ErbB3 or anti-γH2AX antibody for 1 h at 37°C. After washes, cells were incubated with anti-rabbit Alexa Fluor 488 for 30 min at 37°C and mounted on slide holder using mountant medium with DAPI (Prolong gold antifade reagent with DAPI, Life technologies, Gaitherburg, MD, USA). Images were taken at 63X magnification by fluorescent microscope (AxioScope A1, Zeiss).

### Clonogenic and anchorage-independent growth assays

Single cell suspensions were plated at 150–200 cells/well in 6 wells plate for clonogenic assay or 6000cells/well in 24 wells plate on a top layer with 0.4% agar, in the presence or absence of different concentrations of A3 and/or vorinostat or VPA. After indicated days, colonies were visualized by incubation with 0.5% crystal violet dissolved in 20% methanol for 30 min or 5 mg/ml of MTT for 4 h. Colonies were counted using Image-Pro-Plus (Immagini and Computer, Bareggio, Milan, Italy).% of colonies formation was defined as (colony of treated cells/colony of untreated cells) *100. Images were taken at 10X magnification by contrast microscope (Axiovert 40 CFL, Zeiss).

### Spheroid-forming assay

Cells were plated as single cells at 1500–2000 cells/well in ultralow attachment 96-well plates and were grown in sphere medium [23] with or without escalation doses of VPA and with fixed doses of A3, as indicated in the figure, from 3 to 7 days. Spheroids were imaged at 3 and 7 days starting from day 0 of treatment by phase contrast microscope, using a 10× magnification. The formed spheroids after 3 days treatment were also counted using a phase contrast microscope (Axiovert 40 CFL, Zeiss).

### 3D cell viability assay

Approximately 300 cells were seeded in appropriate Gravity plus and Gravity Trap 96 well/plates, following manufacture's recommendations (InSphero, AG, Schlieren, Switzerland) and treated for 72 h with drugs at indicated doses. Cell viability was measured using CellTiter-Glo (Promega, Madison, Wi, USA). On the bases of microscope images, dimensions of ellipsoids were determined by ImageJ software (National Institute of Health, USA) and spheroids volume was calculated applying the modified ellipsoid formula (*π*/6) × *AB*^2^, where *A* is the longest and *B* is the shortest perpendicular axis of an assumed ellipsoid corresponding to spheroid volume.% of spheroid volume was defined as (volume of treated cells at indicated time – volume of treated cells at T0)/(volume of untreated cells at indicated time – volume of untreated cells at T0) * 100.

### Statistics

The results of the antiproliferative assays are expressed as the average of for at least three independent experiments done in quadruplicate, and the standard deviations (SD) as indicated. Representative results from a single western blotting experiment are presented; additional experiments yielded similar results. Real-time PCR data are representative from a single experiment performed in triplicate, ± SD. Similar results were obtained from three different experiments. Immunofluorescence results obtained by flow cytometry are expressed as the means (± SD) for at least three independent experiments, while the immunofluorescence results obtained by microscopy are representative of a single experiment, additional experiments yielded similar results. The results of the clonogenic and anchorage independent assays, as well as spheroid-forming assay and 3D cell viability, are representative from a single experiment performed in duplicate, ± SD.

Statistical significance of the differences was determined by the two-sided Student's *t* test, using SigmaStat software (Systat Software Inc, San Jose, California, USA).

## SUPPLEMENTARY MATERIALS FIGURES


