# Supplementary material for: Synergistic antitumor activity of histone deacetylase inhibitors and anti-ErbB3 antibody in NSCLC primary cultures via modulation of ErbB receptors expression
Source: Oncotarget. 2016 Feb 4;7(15):19559–74. doi: 10.18632/oncotarget.7195 (PMC4991401; doi:10.18632/oncotarget.7195)
Supplement: Supplementary file 1 [file oncotarget-07-19559-s001.pdf]

Synergistic antitumor activity of histone deacetylase inhibitors and anti-ErbB3 antibody in NSCLC primary cultures via modulation of ErbB receptors expression

Supplementary Materials

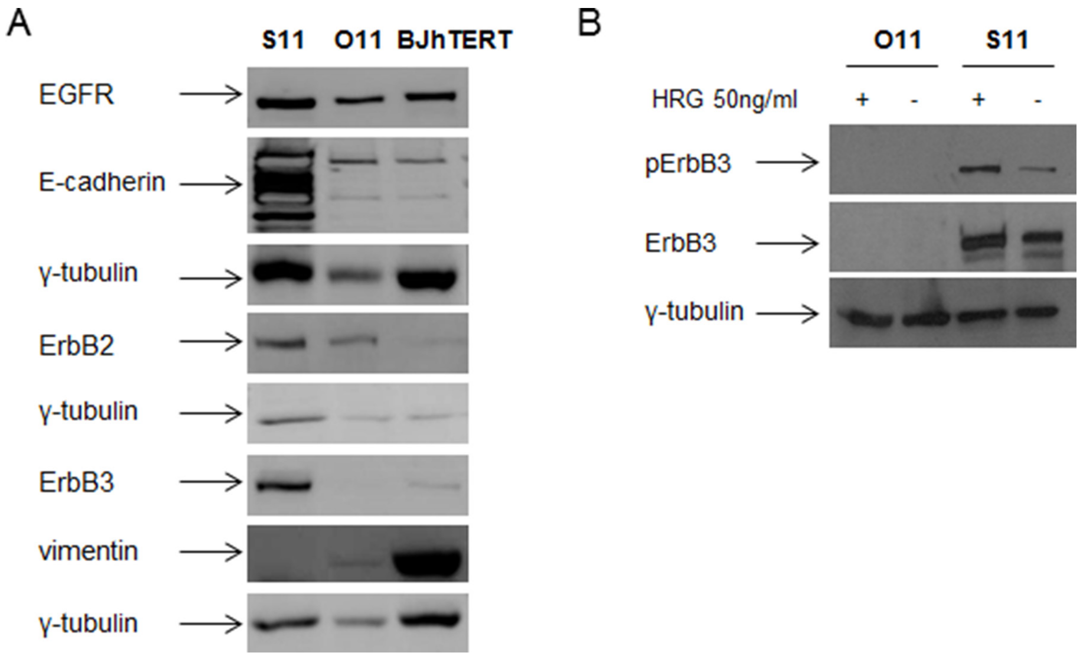

Supplementary Figure S1: S11 and O11 MPE cells showed different expression profile of ErbB receptors and EMT markers compared to normal fibroblast (BJhTERT) cell line. (**A**) Protein lysates from S11, O11 and BJhTERT cells at basal levels, were resolved by SDS-PAGE and immunoblotted with anti- EGFR, ErbB2, ErbB3, E-cadherin and vimentin antibodies. γ-tubulin was used as protein loading control. (**B**) O11 and S11 cells, plated and starved for 24 h, were stimulated with 50 ng/ml of HRG for 10 min. After stimulation, cells were collected, resolved by SDS-PAGE and immunoblotted with pErbB3 and ErbB3 antibodies. γ-tubulin was used as protein loading control.

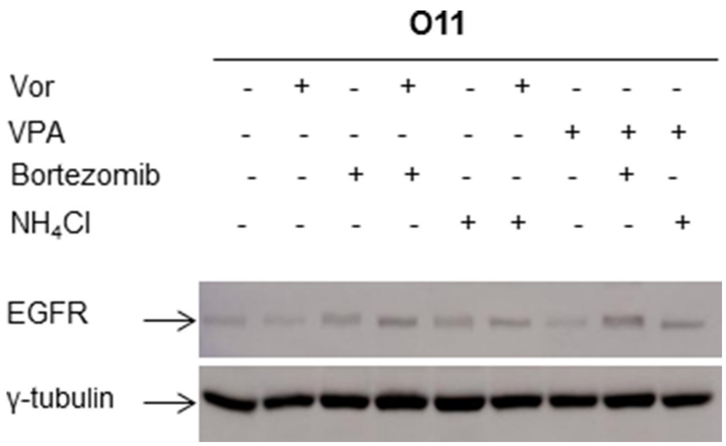

Supplementary Figure S2: Vorinostat and VPA downregulated EGFR expression enhancing either proteasomal or lysosomal receptor degradation in O11 cells. O11 mesenchymal cells were treated with HDACis (vorinostat 1 µM and VPA 0.5 mM) and/or bortezomib 10 nM or NH_4Cl 10 mM at 24 h. Cell extracts were resolved by SDS–PAGE and immunoblotted with anti-EGFR. γ-tubulin immunoblotting indicated the equal loading of samples in each lane.

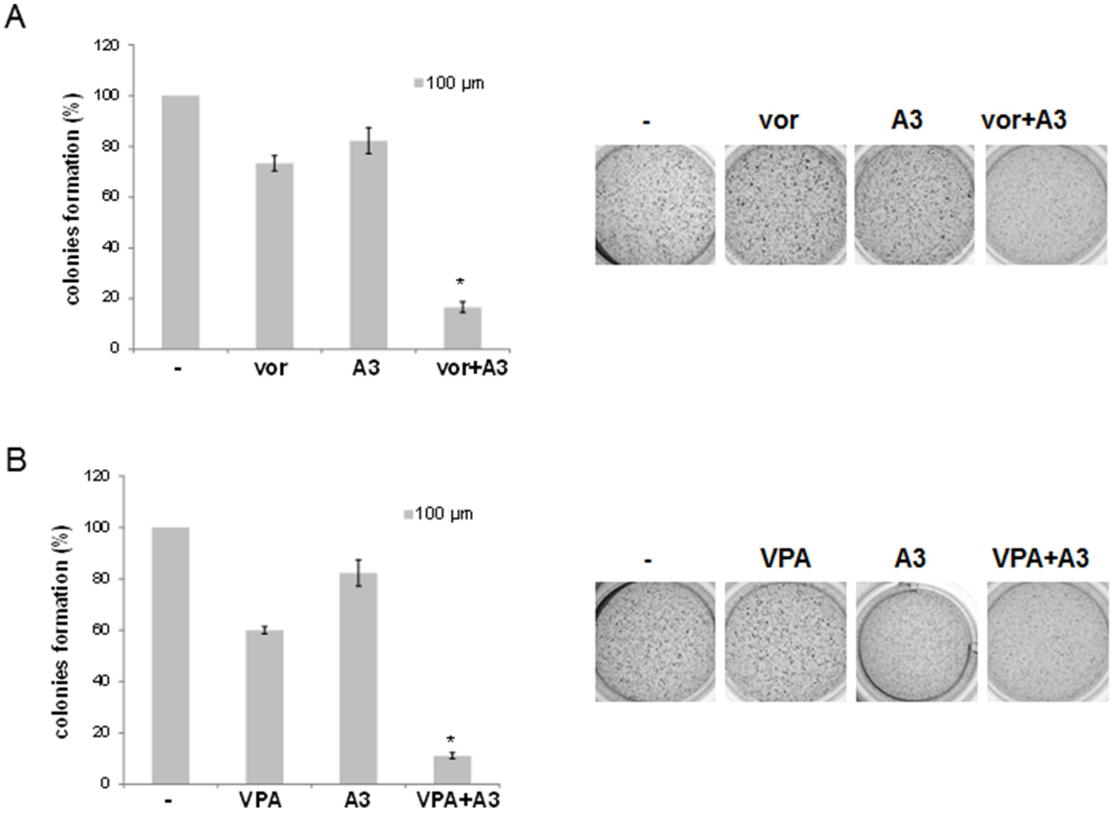

Supplementary Figure S3: Simultaneous treatment with HDACi/moAb A3 induced a synergistic inhibition of S11 cell survival. Anchorage independent growth assays were performed on S11 cells plated, at 6000 cells/well in 24 well/plates, in the absence or presence of drugs at specific doses as follows (**A**) vorinostat: 0.25 µM; (**B**) VPA: 7.5 mM and (A–B) A3: 2.5 µg/ml). After 14 days from seeding, colonies were visualized and counted by MTT colorimetric assay. Colonies formation was expressed as % of control. Statistical analysis for combination vs single agent treatment is reported (*$p < 0.05$ combination vs single agent).

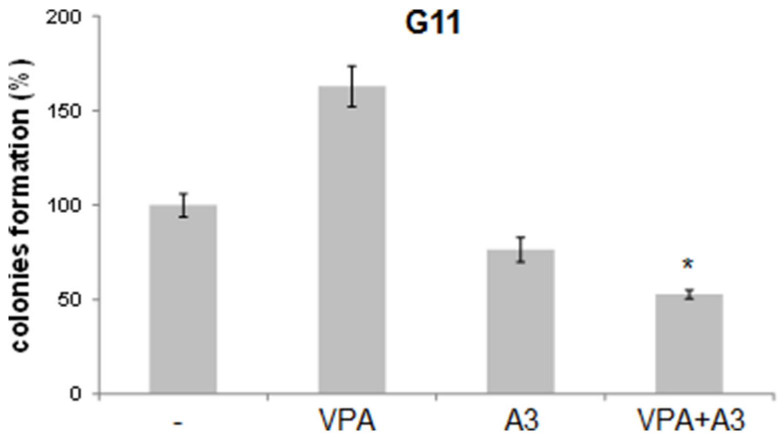

Supplementary Figure S4: Sequential-schedule VPA/moAb A3 treatment induced a strong anti-proliferative effect on mesenchymal G11 cell line. Anchorage independent growth assays were performed on G11 cells plated at 6000 cells/well in 24 well/plates and untreated or treated with VPA 0.5 mM, followed, with 24 h of delay, by A3 1 µg/ml. After 14 days from seeding, colonies were visualized and counted by MTT colorimetric assay, as described in Materials and Methods section. Colonies formation was expressed as % of control. Statistical analysis for combination vs single agent treatment is reported (*$p < 0.05$ combination vs single agent).
